# Concurrent predictors of mathematics achievement for 9-year-old children with Williams syndrome

**DOI:** 10.1038/s41598-024-52639-7

**Published:** 2024-01-30

**Authors:** Vitor N. Guimaraes, Carolyn B. Mervis

**Affiliations:** Department of Psychological and Brain Sciences, University of Louisville, 317 Life Sciences Building, Louisville, KY 40292 USA

**Keywords:** Genetics, Psychology

## Abstract

Research on mathematics achievement by children with Williams syndrome (WS) has been very limited. We describe the math achievement of 72 9-year-olds with WS, compare their math and reading achievement, and explore concurrent predictors of math achievement using the Wechsler Individual Achievement Test-III (WIAT-III) to measure achievement and the Differential Ability Scales-II (DAS-II) to measure cognitive abilities. For both Numerical Operations and Math Problem Solving, mean standard scores (SSs) were in the mild disability range with a full range from severe disability to average ability. Bayesian robust estimation indicated decisive evidence that average reading performance was higher than average math performance. Bayesian multiple linear regression models with informative priors accounted for 60% of the variance in Numerical Operations SS with decisive evidence supporting an effect of Working Memory SS and strong evidence for Nonverbal Reasoning SS and Spatial SS and 71% of the variance in Math Problem Solving SS with decisive evidence supporting effects of Working Memory and Nonverbal Reasoning SS, strong evidence for Verbal SS, and substantial evidence for Spatial SS. These predictors are consistent with those for typically developing individuals, supporting consideration of interventions that have been highly effective for children with math difficulties in the general population.

## Introduction

Functional math skills—the ability to understand, interpret, and manipulate quantitative information in a variety of contexts—are important for living in the modern world^1^. Math skills in adulthood predict concurrent employability^2,3^. Early math skills predict later socioeconomic status (SES). For example, in a large sample representative of the population of the United Kingdom, math skills at age 7 years predicted SES at age 42 years even after taking into account childhood SES, IQ, reading ability at age 7 years, and total years of education^4^. Furthermore, a meta-analysis indicated that math skill at school entry was as good as school-entry reading skill at predicting later reading achievement^5^. Among individuals with intellectual disability (ID), low math achievement relative not only to typically developing individuals but also to individuals with other types of disabilities has been repeatedly documented^6,7^ and is a major challenge for the transition of individuals with ID into adulthood and work settings^8^.

Consistent with previous findings for individuals with ID of mixed etiologies, the few prior studies addressing math achievement for individuals with Williams syndrome (WS)—a rare neurogenetic disorder associated with mild to moderate ID—have reported very low levels of performance (see Van Herwegen and Simms^9^ for a systematic review). The purpose of the present study was to describe the full range of math achievement for a relatively large group of children with WS of the same age (9 years) and to identify not only bivariate relations between math achievement and cognitive abilities but also cognitive predictors that accounted for unique variance in math achievement. In the remainder of the introduction, we first consider the findings of meta-analyses investigating relations between cognitive abilities and math achievement in typically and atypically developing individuals and then describe briefly findings from two large studies examining unique cognitive predictors of math achievement for typically developing children and adolescents. We then review previous studies addressing math achievement in individuals with WS and present the objectives of the current study.

### Cognitive correlates and concurrent predictors of math achievement

Based on a meta-analysis involving 829 correlations, Peng et al.^10^ reported a moderate relation between composite working memory ability and overall math achievement. Separate analyses indicated similar moderate effects for the relation between working memory and either whole number calculation or word-problem solving. On average, working memory ability accounted for about 10% of the variance in math achievement. The relation between working memory and math decreased significantly as chronological age (CA) increased. Strikingly, the relation between working memory and math achievement was significantly stronger for individuals who had math difficulty accompanied by cognitive difficulties such as ADHD or ID than for individuals in the general population, accounting for about 25% of the variance in math achievement. This pattern is consistent with Detterman and Daniel's^11^ finding that correlations between scaled scores on pairs of subtests (including Arithmetic) from the Wechsler Intelligence Scale for Children–revised (WISC-R)^12^ or the Wechsler Adult Scale of Intelligence–revised (WAIS-R)^13^ for the standardization samples were significantly higher for individuals with low estimated IQs (< 78) than for individuals with higher estimated IQs.

In a meta-analysis involving 1129 correlations, Peng et al.^14^ considered the relation between fluid intelligence and math achievement. Overall, the relation was moderate, with stronger effects of nonverbal reasoning (matrix reasoning or non-matrix reasoning) than visuospatial reasoning. Fluid intelligence was significantly more strongly related to word problem solving than to calculation. No significant differences in the strength of relation between fluid intelligence and math achievement were identified as a function of participant type (general population vs. atypical). In this meta-analysis, the atypical group was very broad, including individuals with either developmental (e.g., autism spectrum disorder or learning difficulties/disabilities) or acquired (e.g., brain injury or Alzheimer disease) disorders, and was not broken down further.

In a third meta-analysis involving 2066 correlations, Peng et al.^15^ addressed relations between language ability and math achievement. The correlation with word-problem solving was moderate to strong and was significantly higher than the correlation with calculation, which was moderate. Both correlations were significantly stronger for the general population group than for the atypical group (defined as in Peng et al.^14^). The correlation with calculation (but not with word-problem solving) decreased significantly with age. Partial correlations between language and math achievement controlling for both working memory and fluid intelligence were weak but statistically significant, with working memory and fluid intelligence accounting for 33–42% of the variance in the relation of language with calculation and 48–67% of the variance in the relation of language with word-problem solving.

To consider the unique contributions of broad stratum cognitive abilities to math achievement, Villeneuve et al.^16^ modeled the performance of the > 2000 children included in the co-norming sample of the Kaufman Assessment Battery for Children–second edition^17^ and the Kaufman Test of Educational Achievement–second edition^18^. In the final model for the full sample, statistically significant contributions of nonverbal reasoning and short-term memory (including working memory) were identified for math computation, and statistically significant contributions of verbal knowledge, nonverbal reasoning, and short-term memory were found for math problem solving. Hale et al.^19^ examined the unique and shared contributions of the Differential Ability Scales-II (DAS-II)^20^ clusters to the variance in the Wechsler Individual Achievement Test-II (WIAT-II)^21^ Numerical Operations and Math Reasoning (i.e., problem solving) subtests in the linking sample of 417 typically developing 6- to 17-year-olds. For Numerical Operations, contributions of unique variance ranging from 1.0 to 3.2% and shared variance from 21.6 to 28.4% were reported for the Verbal, Nonverbal Reasoning, Spatial, and Working Memory clusters. For Math Reasoning, a similar pattern was reported for the same clusters, although higher unique (1.8–4.9%) and shared (29.1–36.0%) contributions were found. The authors further noted that the unique contribution of Working Memory was likely underestimated due to shared variance with the Spatial and Verbal clusters. The total proportion of variance accounted for by the linear predictors (*R*^*2*^) was 0.458 for Numerical Operations and 0.579 for Math Reasoning.

### Math achievement in individuals with Williams syndrome

Williams syndrome (WS) is a neurogenetic disorder caused by a microdeletion of 25–27 genes on chromosome 7q11.23^22^, with an estimated prevalence of 1:7500 live births^23^. WS is associated with distinctive physical, behavioral, and cognitive features^22^. Most individuals with WS have mild intellectual disability, but the full range of intellectual functioning is from severe disability to low average or average intellectual ability^24,25^. When considering cognitive abilities separately, individuals with WS typically have relative strengths in verbal reasoning, nonverbal reasoning, verbal short-term memory, and concrete vocabulary accompanied by substantially weaker relational language and visuospatial construction abilities^22,24,26,27^. As is common with rare neurogenetic disorders, there is a paucity of studies examining the academic achievement of individuals with WS, and only a small proportion of these studies addressed math achievement. In the remainder of this section, we summarize their findings.

The earliest reference to math achievement among individuals with WS was provided in one of the first case series published, predating the identification of the syndrome's genetic etiology by almost 30 years. In their classic paper, von Arnim and Engel^28^ included the scaled score on the Arithmetic subtest of the Wechsler Intelligence Scale for Children (WISC)^29^ in two of their four case descriptions. A 13-year-old boy earned a scaled score of 1 [corresponding to a standard score (SS) of 55], and a 10½-year-old boy earned a scaled score of 2 (equivalent to a SS of 60). These scaled scores were lower than the children's scaled scores on the WISC Vocabulary subtest [3 (corresponding to a SS of 65) for the older boy and 4 (corresponding to a SS of 70) for the younger], providing the first formal evidence of stronger language ability than mathematical ability for individuals with WS.

All subsequent studies for which standardized scores were reported also measured math ability using the Arithmetic subtest of a version of the WISC or its adult counterpart, the Wechsler Adult Intelligence Scale (WAIS). Arnold et al.^30^ reported that for a sample of 23 children aged 7–12 years, mean scaled score on the WISC-R^12^ Arithmetic subtest was significantly lower than mean scaled scores on the Comprehension and Picture Completion subtests. Mean scaled scores were not provided. Udwin et al.^31^ reported a mean scaled score of 2.36 (equivalent to a SS of 62) with a *SD* of 1.82 on the WISC-R Arithmetic subtest for 44 children aged 6–15 years (including nine who had participated in the Arnold et al.^30^ study), which was lower than the mean scaled scores on the Vocabulary (4.27, corresponding to a SS of 71) and Similarities (4.50, corresponding to a SS of 72) subtests. Howlin et al.^32^, who tested 62 adults [including 23 who had participated in the Udwin et al.^31^ study as children] reported a mean scaled score of 2.74 (equivalent to a SS of 64) with a *SD* of 1.23 on the WAIS-R^13^ Arithmetic subtest, which was significantly lower than the mean scaled scores for the Vocabulary and Comprehension subtests. Howlin et al.^33^, based on a follow-up study of 47 participants from the Howlin et al.^32^ study, reported a WAIS-III^34^ Arithmetic scaled score of 2.0 (equivalent to a SS of 60). Howlin et al.^33^ also reported a cross-sectional study of 92 adults aged 19–55 years with genetically-confirmed WS. For this sample, the mean WAIS-III Arithmetic scaled score was approximately 1.75 (corresponding to a SS of 59); the *SD* was not reported for either sample. In summary, the performance of individuals with WS on the Arithmetic subtest was consistently characterized by very low mean scaled scores and limited variability both relative to other abilities assessed for the same individuals and when compared to the performance of individuals in the general population. Although it is clear that the lowest Arithmetic scaled score in each study was 1, none of the studies indicated what the highest Arithmetic scaled score was.

In three other studies, age equivalents (AEs), grade equivalents (GEs), or raw scores that could be converted to AEs using the assessment test manual were reported rather than standardized scores. Van Herwegen et al.^35^ reported a mean raw score of 8.63 (*SD* = 3.91, range: 0–17) on the Wechsler Individual Achievement Test-II (WIAT-II)^21^ Numerical Operations subtest, a measure of math computation, for 24 individuals with WS aged 8–51 years. Based on the WIAT-II technical manual, the mean raw score corresponded to an AE of 6 years 4 months; AEs ranged from 4 years 0 months to 8 years 4 months. Pagon et al.^36^ reported that for eight of nine individuals aged 10–20 years, GEs ranged from 0.3 (= K.3) to 1.8 on the Mathematics subtest of the Peabody Individual Achievement Test^37^. The remaining child, aged 14 years 8 months, earned a GE of 3.8. The Mathematics GEs were lower than the GEs on the Reading Recognition, Reading Comprehension, and Spelling subtests for all but one participant, who earned a GE of 0.5 on all four subtests. O'Hearn and Landau^38^ indicated that AEs on the Test of Early Mathematics Ability-2 (TEMA-2)^39^ for 13 of 14 participants (aged 10–38 years; mean = 17.75 years) ranged from 5 years 6 months to 7 years 10 months (median = 7 years 0 months). The remaining participant, aged 13 years, did not reach ceiling, resulting in a reported AE of > 8 years 11 months (the oldest age for which the TEMA-2 was normed) and providing further evidence that some individuals with WS are able to acquire math skills beyond the second-grade level.

The relation between math achievement and cognitive abilities for individuals with WS has been addressed in two studies. O'Hearn and Landau^38^ reported statistically significant and strong correlations between TEMA-2 raw score and raw scores from both the Verbal (*r* = 0.54) and Matrices (*r* = 0.77) components of the Kaufman Brief Intelligence Test^40^. Van Herwegen et al.^35^ reported a statistically significant and strong correlation (*r* = 0.71) between raw scores on the WIAT-II Numerical Operations subtest and Raven's Coloured Progressive Matrices^41^, a measure of nonverbal reasoning. The correlation between WIAT-II Numerical Operations raw score and British Ability Scales–second edition^42^ Pattern Construction ability score, a measure of Spatial ability, was moderate (*r* = 0.36) but not statistically significant.

### Current study

The purpose of the present study was to describe math achievement, how it compared to reading achievement, its relations to measures of cognitive functioning, and its cognitive predictors for a relatively large sample of children with genetically confirmed classic-length WS deletions in a narrow age range. Age 9 years was selected, since it is the youngest age at which an individual achievement test was routinely administered to participants in the senior author's lab. For all variables included in the inferential analyses, age-based SSs were used. SSs are a continuous measure of relative standing whose values indicate the position of an individual within a continuum of performance derived from a reference sample representative of the general population—the norming sample. We chose to compare children’s performance to the norming samples for the assessments used rather than to a control group of typically developing children tested in our lab for two primary reasons. First, as described in the Participants section, our sample was from across the US and from rural, suburban, and urban communities so the norming samples, which were stratified by region and by community size and which included both typically developing children and children with disabilities, were considerably more representative of the general population of 9-year-olds than a sample of local typically developing children would have been. Second, by devoting our resources to testing only children with WS, we were able to assess a considerably larger sample of children with WS, making our findings much more likely to generalize to the population of children with WS.

Four research questions were addressed:

***Question 1.*** What are the average SSs and *SD*s and the full range of performance on standardized math achievement measures for 9-year-olds with WS?

As described earlier, results of past studies using standardized assessments suggest that individuals with WS have low math achievement with limited variability. To address this question, we used SSs to assess two components of math achievement: computation and word-problem solving.

***Question 2.*** How does performance on the two math achievement components compare to each other and to reading achievement in 9-year-olds with WS?

Based on unpublished data from an independent sample of children with WS who were administered a previous edition of the achievement test used in the current study in our lab (see Supplementary Materials Appendix A, Table S1), as well as the fact that word problem solving requires coordination of computational skills with additional skills (e.g., listening comprehension, verbal reasoning, and spatial reasoning), we hypothesized that average performance on the math computation measure would be higher than average performance on the math problem solving measure.

With respect to comparisons between math and reading achievement, findings from the single prior published study^36^ for which reading and math achievement were measured on the same standardized instrument suggest that the reading abilities of individuals with WS are more advanced than their math abilities. In addition, the reading SSs of the independent sample of children with WS described in the Supplementary Materials were considerably higher than their math SSs (Appendix A, Table S1). Based on these findings, we hypothesized that the average SSs on the math achievement measures would be lower than the average SSs on the reading achievement measures.

***Question 3.*** What is the relation between math achievement and cognitive abilities in 9-year-olds with WS?

O'Hearn and Landau^38^ and Van Herwegen et al.^35^ both found that math achievement for individuals with WS was significantly related to nonverbal reasoning ability. In addition, O'Hearn et al.^38^ reported a statistically significant relation between math achievement and verbal reasoning ability, and although Van Herwegen et al.^35^ did not find a statistically significant relation with spatial ability, the reported correlation was moderate, for a sample size that was about one-third the size of the present sample. Results from the meta-analyses discussed above^10,14,15^ indicated that both computation and word-problem abilities were moderately related to verbal, nonverbal reasoning, spatial reasoning, and working memory abilities for both typically developing and atypically developing individuals. Based on these findings, we expected to find the same relations for children with WS.

***Question 4.*** What are the cognitive predictors of math achievement in individuals with WS?

To the best of our knowledge, no past studies have examined the unique cognitive predictors of math achievement in individuals with WS. The Hale et al.^19^ results for children in the general population were the most informative for the present study, since the cognitive measure used in that study (DAS-II)^20^ is the same as the one used in the present study and the math achievement measure used (WIAT-II)^21^ is an earlier version of the math achievement measure used in the present study (WIAT-III)^43^. To integrate the information from Hale et al.^19^ directly into our models, Bayesian analyses were selected. Below, we describe our hypotheses for the two math achievement domains considered.

Based on Hale et al.'s^19^ findings, we expected to find substantial evidence indicating unique contributions to the variance in both math computation and math problem solving from the DAS-II Verbal, Nonverbal Reasoning, Spatial, and Working Memory clusters. Based on Detterman and Daniel's^11^ finding that correlations among pairs of WISC or WAIS subtests, including Arithmetic, were considerably higher for individuals with lower IQs than for individuals with higher IQs and Peng et al.'s^14^ similar finding that the meta-correlation between working memory and math achievement was significantly higher for individuals with math difficulty combined with cognitive difficulties than for individuals in the general population, we expected that the proportion of variance explained by the predictors (Verbal, Nonverbal Reasoning, Spatial, and Working Memory cluster SSs) would be at least as high as that reported by Hale et al.^19^ (0.458 for math computation and 0.579 for math problem solving) and that the contribution of Working Memory would be greater than Hale et al.^19^ reported.

## Method

### Participants

The final sample included 72 participants (35 girls, 37 boys) with genetically confirmed classic-length deletions of the WS region aged 9.01–9.89 years (*M* = 9.32 years, *SD* = 0.27). All participants were native speakers of English. Participants' median school grade was third, with an interquartile range from second to third grade. Two (2.8%) children were tested the summer after they completed first grade. Of the remaining participants, 25 (34.7%) were in or had recently completed second grade, 41 (56.9%) were in or had recently completed third grade, and four (5.6%) were in fourth grade.

Participants were from 23 US states representing all US census regions (23.6% Northeast, 40.3% South, 22.2% Midwest, 11.1% West) and two Canadian provinces (2.8%). Participants' racial/ethnic background was 77.7% White non-Hispanic, 9.7% White Hispanic, 1.4% Black/African American non-Hispanic, 5.6% multiracial non-Hispanic, and 5.6% multiracial Hispanic. One additional child with a classic-length deletion was excluded from the final sample because he was nonverbal. Data collection began in December 2009 and ended in February 2020.

### Measures

All measures have a general population mean of 100 and *SD* of 15. All internal consistency measures reported are for 9-year-olds in the norming sample, as described in the assessments' technical manuals.

### Cognitive abilities

Cognitive abilities were measured by the School Age battery of the Differential Ability Scales–second edition (DAS-II)^20^, an individually administered standardized assessment. Subtest-yielded scores contribute to clusters, each intended to be a measure of broad-stratum cognitive abilities according to the Cattell-Horn-Carroll model (CHC). Reported internal consistency estimates are IRT-based.

#### Verbal ability

Verbal ability was measured by performance on the Verbal cluster. This cluster includes two subtests: Word Definitions, which measures vocabulary and semantic knowledge; and Verbal Similarities, which measures verbal reasoning. The CHC broad-stratum ability measured by this cluster is crystallized knowledge (*Gc*). Internal consistency for this cluster is 0.90.

#### Nonverbal reasoning ability

Nonverbal reasoning ability was measured by performance on the Nonverbal Reasoning cluster. This cluster contains two subtests: Matrices, a matrix reasoning measure; and Sequential and Quantitative Reasoning, a nonverbal measure of relational reasoning. The items from the latter subtest that are administered to 9-year-olds measure sequential reasoning rather than quantitative reasoning. The CHC-based ability measured by this cluster is fluid reasoning (*Gf*). Internal consistency for this cluster is 0.94.

#### Spatial ability

Spatial ability was measured by performance on the Spatial cluster. This cluster contains two subtests: Recall of Designs, which measures visual short-term memory for spatial relations as assessed by the child's drawings of abstract patterns from memory; and Pattern Construction, which measures visual-perceptual matching (including spatial orientation) and construction as assessed by copying pictured 2-block or 4-block patterns using cubes with patterned sides. Children who cannot copy at least three of these patterns accurately are shown pictured patterns composed of 2, 4, or 6 solid black or yellow squares and asked to copy them using rubber squares that are yellow on one side and black on the other. As suggested by the name, the CHC broad-stratum factor measured by this cluster is visual-spatial ability (*Gv*). Internal consistency for this cluster is 0.94.

#### Working memory

Working memory was measured by performance on the Working Memory cluster, which includes two subtests. Recall of Digits—Backward measures verbal working memory. In this subtest, the child must repeat strings of digits stated by the examiner in the reverse order. Recall of Sequential Order requires the coordination of visuospatial working memory and verbal working memory. For the items administered to 9-year-olds, the child must repeat a sequence of human body parts stated by the examiner while rearranging the items from highest to lowest on the body. Early items provide visual support in the form of a picture of a child; for later items, visual support is not provided. The CHC broad-stratum ability measured in this cluster is short-term memory (*Gsm*), which includes what is more generally known as working memory. Internal consistency for this cluster is 0.94.

#### Overall cognitive ability

Overall cognitive ability was measured by the General Conceptual Ability composite (GCA; similar to IQ) and was reported for descriptive purposes only. The GCA is based on performance on the subtests included in the Verbal, Nonverbal Reasoning, and Spatial clusters. Internal consistency for this composite is 0.97.

### Math achievement

Math achievement was measured by performance on two subtests from the Wechsler Individual Achievement Test–third edition (WIAT-III)^43^, an individually administered standardized assessment of academic achievement. For descriptive purposes, we also report performance on the Mathematics Composite, which is based on performance on the two subtests that were administered. Split half internal consistency estimates are reported.

#### Computation

Computation was measured by performance on the Numerical Operations subtest. The items administered to the children in this study assess counting, recognition of basic mathematical symbols, and performance of basic arithmetic operations. Items are presented in a booklet as equations for the child to solve and then write the answer. Following the standardization rules, testing was terminated after four consecutive incorrect responses. Internal consistency for this subtest is 0.90.

#### Word problem solving

Word problem solving was measured by performance on the Math Problem Solving subtest. For most of the items that were administered to the participants in this study, the examiner shows the child a picture and then asks a question about the picture that requires application of the child's mathematical knowledge. The child responds verbally. Scrap paper is available for the child to use if desired. The abilities measured include enumerating sets of objects, simple arithmetic, practical math (e.g., relational concepts, time, money), geometry, algebra, and statistical reasoning. Following the standardization rules, testing was terminated after four consecutive incorrect responses. Internal consistency for this subtest is 0.87.

### Reading achievement

Reading achievement was measured by the Basic Reading Composite and the Reading Comprehension subtest of the WIAT-III. Split half internal consistency estimates are reported for all measures.

#### Basic or word reading skill

Single word reading skill was measured by the Basic Reading Composite. This composite includes two subtests: Word Reading, which measures real word reading skill; and Pseudoword Decoding, which measures non-word reading skill. Internal consistency is 0.98 for each subtest and 0.99 for the Basic Reading Composite.

#### Reading comprehension

Reading comprehension was measured by the Reading Comprehension subtest. Participants were presented with short passages in a booklet which they could read silently or aloud at their discretion. Reading errors were not corrected. After reading each passage, the participant was asked four to eight open-ended questions by the examiner. The passage was visible to the child during the question period. Internal consistency for this subtest is 0.80.

### Procedure

The study protocol was reviewed and approved by the University of Louisville Institutional Review Board. All procedures were performed in accordance with the ethical standards of this Institutional Review Board and with the Declaration of Helsinki. Parents or legal guardians of all participants provided written informed consent and participants provided oral assent. All measures were individually administered by trained examiners at a single US university as part of a broader 2-day assessment. The standardized procedures for administration and scoring were followed. Scoring of all assessments was checked by a second examiner and the rare disagreements between examiners were resolved by consensus or consultation with the senior author.

### Data analysis

#### Group-level comparisons among achievement measures

To compare group-level differences between the two domains of math achievement and between math achievement and reading achievement, we used Bayesian robust estimation. To represent our prior knowledge about performance on computation and word problem solving, we used the mean SSs and *SD*s obtained from an independent sample of 45 children with WS who completed the WIAT-II Numerical Operations and Math Reasoning subtests (see Supplementary Materials Appendix A and Table S1) as the best estimates of expected performance on the WIAT-III Numerical Operations and Math Problem Solving subtests. For performance on the reading achievement subtests, we used the results reported by Mervis et al.^44^ for a sample of 70 9-year-olds with WS, all of whom were included in the present study. In particular, the distributions of differences between SSs on each pair of variables used in the pairwise comparisons were calculated using the difference between the mean values mentioned above, and the respective variances were calculated after removing the covariance between the two variables being compared. Full descriptive statistics for the sampled prior distributions are provided in Supplementary Materials (Appendix B, Table S2).

In addition to determining the pattern of group-level differences in relative performance on the math and reading measures for the full sample, we conducted a cluster analysis to explore the possibility of the existence of subgroups of participants who showed different profiles of relative strengths and weaknesses. The cluster analysis was conducted in two steps. First, we performed a hierarchical clustering based on Euclidean distances and applied Ward’s *D*^*2*^ criterion to arrive at the optimal number of clusters. Based on Ward’s *D*^*2*^ values, the optimal number of clusters was between three and five. The five-cluster option was rejected to keep our results as parsimonious as possible, leaving the three- and four-cluster options as candidates for soft clustering. After conducting soft clustering, the similarity indices (ranging from 0 to 1) for all participants were calculated based on both options. To approximate the hard clustering needed for the subsequent analyses, participants were assigned to the cluster where they obtained the highest similarity score. The four-cluster option was selected after inspection of the SSs obtained by individuals in each cluster. Within-cluster descriptives and individual-level discrepancies (like those calculated for the overall sample using critical values reported in the WIAT-III manual) were then computed.

A second step in the cluster analysis involved comparing adjacent clusters with the objective of identifying whether the predicted patterns of stronger performance on math computation compared to math problem solving and stronger reading achievement compared to math achievement held across clusters. In this step, a Bayesian multivariate linear model with the WIAT-III Numerical Operations, Math Problem Solving, Basic Reading Composite, and Reading Comprehension SSs as response variables and the clusters as predictors was performed. In addition, the respective distributions of standardized mean differences were computed. Qualitative interpretation of the point-estimates from these distributions followed Sawilowsky’s^45^ categories: 0.20 = small, 0.50 = medium, 0.80 = large, and 1.20 = very large standardized difference. Since no prior information about the distributions of differences between clusters was available or could be derived from the literature, uniform priors were used. Descriptive statistics for all sampled prior distributions are provided in the Supplementary Materials (Appendix B, Table S2).

#### Individual-level comparisons among achievement measures

At the individual level, we computed the frequency of discrepancies between pairs of SSs on the achievement measures taking into account the direction of the discrepancy and whether or not the difference was statistically significant at the *p* < 0.05-level based on the critical values reported in the WIAT-III technical manual^43^ for ages 4.00–11.99 years. In the manual, critical values are reported with two significant digits. We rounded all critical values to the next highest whole integer to avoid overestimation of the occurrence of statistically significant discrepancies. The WIAT-III technical manual does not report critical values for a discrepancy between the Basic Reading Composite and the two math subtests. However, critical values for comparisons between each of the math subtests and each of the reading subtests included in the Basic Reading Composite (Word Reading and Pseudoword Decoding) were available. As all of these were between 8.0 and 8.9, we used 9 as the critical value for the comparisons with Basic Reading Composite. These individual-participant discrepancies are reported first for the sample as a whole and then separately for the individuals as a function of cluster.

#### Regression analyses

Correlations among the variables used in the regression analyses were computed using bivariate Bayesian Pearson product-moment correlations with a weakly informative beta prior. Because data collection spanned several years, correlations with year of testing using the same weakly informative beta prior were also run for descriptive purposes. Full descriptive statistics for the sampled prior distributions are provided in the Supplementary Materials (Appendix B, Table S2). Qualitative interpretation of point estimates of the posterior distribution of correlations followed Cohen's^46^ classical categories: 0.10 = weak, 0.30 = moderate, and 0.50 = strong.

Two Bayesian multiple linear regressions were performed with WIAT-III Numerical Operations SS and Math Problem Solving SS as dependent variables and DAS-II Verbal, Nonverbal Reasoning, Spatial, and Working Memory SSs as predictors. For both regression analyses, a prior was set on the hypothesized distribution of multiple correlation coefficients using the values reported by Hale et al.^19^ as the lower bound of credibility. The reference multiple correlation was 0.458 for the Numerical Operations model and 0.579 for the Math Problem Solving model.

We set Student *t* priors for the dependent variables using the results from the independent sample reported in the Supplementary Materials (see Table S1) for WIAT-II Numerical Operations and Math Reasoning SSs. For the WIAT-III Numerical Operations SS model, the prior mean was 60.89 and *SD* was 15.49; for the WIAT-III Math Problem Solving SS model, the prior mean was 57.00 and *SD* was 15.16. For both priors, degrees of freedom were set at 30 to better approximate a Gaussian distribution. The variance parameters were centered and truncated at 0 with *SD* = 15 (the general population *SD* of all measures used in the analyses) and degrees of freedom = 3. All descriptive statistics for the priors distributions used in the regression analyses are provided in the Supplementary Materials (Appendix B, Table S2).

#### Assessing effects

We planned to make discrete judgments about effect existence for group-level comparisons, comparisons between adjacent clusters, bivariate correlations, and predictors in the regression analyses. Bayes factors (BFs) expressed on a base 10 scale were used for all our decisions except for the cluster comparisons. Qualitative assessment of *BF*s was based on the thresholds suggested by Kass and Raftery^47^ as follows: *BF* < 1 = evidence supports the null hypothesis; 1 ≤ *BF* < 10^1/2^ (i.e., 3.16) = negligible, 10^1/2^ ≤ *BF* < 10^1^ = substantial, 10^1^ ≤ *BF* < 10^2^ = strong, and *BF* ≥ 10^2^ = decisive evidence for the experimental hypothesis. In addition, we report support intervals indicating the values of the posterior distribution that predicted the observed data better than the average by a Bayes factor of 3.16 (= 10^1/2^), the threshold for substantial evidence in favor of the experimental hypothesis.

For the cluster comparisons, the probability of direction was computed for each comparison between adjacent clusters. Using the Dunn–Šidák procedure, the acceptance threshold was changed from the typical 0.9750 (equivalent to a Frequentist *p* value of 0.05, two-tailed) to 0.9875 (equivalent to a Frequentist *p* value of 0.025, two-tailed) to adjust for the addition of two post-hoc comparisons. In other words, the hypothesis that any two clusters differed in each achievement measure was accepted if the probability of direction was higher than 0.9875.

## Results

### Average levels of functioning

The average level of overall intellectual functioning, as measured by the DAS-II GCA, was within the mild intellectual disability range (*M* = 65.00, *SD* = 11.41, range: 41–95). Descriptive statistics for performance on the DAS-II clusters and WIAT-III mathematics and reading measures are presented in Table 1. Consistent with prior findings for larger samples of children with WS^24^, average performance was in the borderline range for the Verbal and Nonverbal Reasoning clusters. Average performance on the Spatial cluster was at the lower level of the mild disability range and considerably lower compared to performance on the Verbal cluster (*M*_difference_ = 16.53, *SD* = 2.34, BF = 3.16 *SI* for *M* [13.85, 19.07], *BF*_*10*_ = 7.49 × 10^5^) and the Nonverbal Reasoning cluster (*M*_difference_ = 22.50, *SD* = 2.15, BF = 3.16 *SI* for *M* [19.94, 24.94], *BF*_*10*_ = 2.56 × 10^10^).

**Table 1:** Descriptive statistics for the variables included in the regression analyses and group-level comparisons among mathematics and reading measures.

Measure	Mean	Median	*SD*	Range
DAS-II				
Verbal SS	71.38	73.50	14.43	31^a^–103
Nonverbal Reasoning SS	77.36	75.50	12.41	49–103
Spatial SS	54.92	54.50	13.72	32^a^–90
Working Memory SS	62.92	66.00	17.74	33^a^–101
WIAT-III—Mathematics				
Numerical Operations SS	64.35	67.00	15.33	40^a^–107
Math Problem Solving SS	57.89	58.00	13.65	40^a^–96
WIAT-III—Reading				
Basic Reading Composite	73.79	73.00	12.68	52^b^–106
Reading Comprehension SS	68.46	69.00	17.92	40^a^–110

*N* = 72. *DAS-II* Differential Ability Scales-II, *SS* standard score, *WIAT-III* Wechsler Individual Achievement Test-III.

^a ^Lowest possible standard score. ^b ^Lowest possible standard score at age 9 years.

Average performance on the Working Memory cluster was in the middle of the mild disability range. For both mathematics subtests, performance was typically in the mild disability range. Average performance on the Mathematics Composite also was in the mild disability range (*M* = 60.93, *SD* = 13.47, range: 40–102). Performance for the reading measures [previously reported in Mervis et al.^44^ for 70 of the 72 children in the current sample] typically was in the borderline range for single word reading and the top of the mild disability range for reading comprehension. For all measures, there was considerable variability, with the full range of performance from moderate or severe disability to average for the general population.

The period of data collection was necessarily quite long for this study in order to obtain a reasonably large sample of children with WS who were very similar in age. To address the possibility that SSs on the DAS-II and/or the WIAT-III shifted systematically over time, we correlated each child’s SSs for each of the measures used in the analyses with the year in which the child was tested. For the DAS-II measures, correlations with year of testing ranged from *r* = −0.09 (*BF*_*10*_ = 0.360, negligible evidence for the null hypothesis) for Nonverbal Reasoning SS to *r* ≤ 0.01 (*BF*_*10*_ = 0.269, substantial evidence for the null hypothesis) for Spatial SS. For the WIAT-III measures, correlations with year of testing ranged from *r* = −0.09 (*BF*_*10*_ = 0.361, negligible evidence for the null hypothesis) for Math Problem Solving SS to *r* = 0.02 (*BF*_*10*_ = 0.271, substantial evidence for the null hypothesis) for Numerical Operations SS. These correlations are all very small and include both positive and negative values, suggesting that collecting the data over a relatively long period of time is unlikely to have affected our findings.

There are serious psychometric concerns regarding AEs. For example, AEs are not on an interval scale, AEs from one measure cannot be directly compared to AEs from any other measure—even if the measures were normed on the same sample, and AEs that are quite discrepant from a child's CA may nonetheless correspond to performance that would be considered typical for a child of that CA^48–50^. However, as AEs or GEs rather than SSs were provided^36,38^ or were the only measure that could be computed from the raw scores^35^ in the three prior studies in which a standardized assessment of math achievement was administered to individuals with WS, nonparametric descriptive statistics for the present participants' AEs on the WIAT-III Numerical Operations and Math Problem Solving subtests are provided in Table S3 for comparison, along with the corresponding nonparametric descriptive statistics for their SSs.

### Group-level comparisons

Group-level pairwise comparisons were performed via Bayesian robust estimation (see Table 2). There was decisive evidence in support of performance (SS) on the Numerical Operations subtest being, on average, better than performance on the Math Problem Solving subtest, compared to a null hypothesis covering negative values and zero. There also was decisive evidence in support of performance on Basic Reading Composite being better than performance on either Numerical Operations or Math Problem Solving. Finally, comparisons with Reading Comprehension indicated decisive evidence of stronger performance than on Math Problem Solving and substantial evidence of stronger performance than on Numerical Operations.

**Table 2:** Pairwise group comparisons via robust estimation of discrepancies between standard scores on WIAT-III mathematics and reading measures.

Comparison			Posterior estimate		BF = 3.16 *SI* for *M* Diff.		Bayes factor (*BF*_*10*_)	
A	B	Direction	*M* Diff.	*SD*	LL	UL	Estimate	Strength^a^
NOP	MPS	A > B	6.35	2.38	4.17	8.53	1.57 × 10^2^	+++
	BRC	A < B	9.54	2.32	6.90	12.04	4.97 × 10^3^	+++
	RC	A < B	4.20	2.75	1.18	7.15	7.23	+
MPS	BRC	A < B	15.92	2.18	13.49	18.30	2.46 × 10^7^	+++
	RC	A < B	10.60	2.62	8.08	13.28	2.37 × 10^3^	+++

*N* = 72. *WIAT-III* Wechsler Individual Achievement Test-III, *M Diff.* mean difference, *SI* support interval.

*NOP* WIAT-III Numerical Operations standard score (SS), *MPS* WIAT-III Math Problem Solving SS, *BRC* WIAT-III Basic Reading Composite SS, *RC* III Reading Comprehension SS.

^a ^+ = substantial, +++ = decisive evidence for the experimental hypothesis.

To consider the possibility that the sample contained one or more subgroups for which the full-sample findings of relative strengths and weaknesses were not representative, a cluster analysis (as described in “Data analysis”) was performed, yielding four clusters.

**Figure 1:**
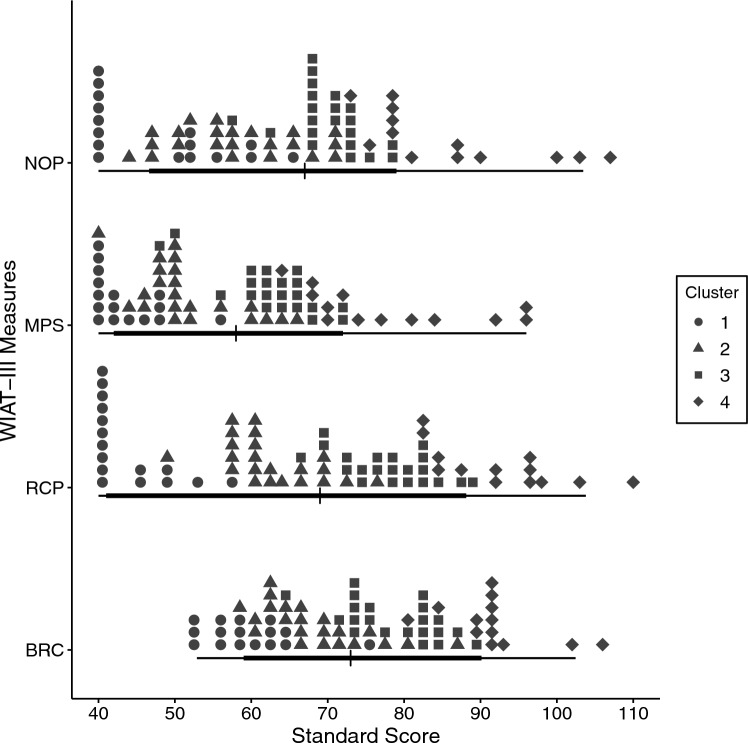
Distributions of standard scores on the Wechsler Individual Achievement Test-III (WIAT-III) Numerical Operations (NOP) subtest, Math Problem Solving (MPS) subtest, Reading Comprehension (RCP) subtest, and Basic Reading Composite (BRC) as a function of cluster. The thin bar under each box plot indicates the 95% confidence interval for the standard score, the thick bar indicates the 75% confidence interval, and the vertical line in the thick bar indicates the median.

The full distribution of SSs earned by the 72 participants is shown in Fig. 1 as a function of achievement measure and Cluster, and the descriptive statistics for SSs on the math and reading measures are presented separately for each cluster in Table 3. As shown in this table, Cluster 2 evidenced the same pattern as the full sample: mean SS was highest for Basic Reading Composite, followed by mean Reading Comprehension SS, then mean Numerical Operations SS, and lowest for mean Math Problem Solving SS. The remaining clusters all showed the same pattern as the full sample for differences among Basic Reading Composite SS, Numerical Operations SS, and Math Problem Solving SS. However, the discrepancies between Reading Comprehension SS and mean performance on the other three achievement measures varied. For Cluster 1, mean Reading Comprehension SS was lower than mean Numerical Operations SS and about the same as mean Math Problem Solving SS; and for Clusters 3 and 4, mean Reading Comprehension SS was about the same as mean Basic Reading Composite SS.

**Table 3:** Descriptive statistics as a function of cluster for the WIAT-III measures included in the cluster analysis.

Cluster	WIAT-III Mathematics						WIAT-III Reading					
	Numerical Operations SS			Math Problem Solving SS			Basic Reading Composite			Reading Comprehension SS		
	*M*	*SD*	Range	*M*	*SD*	Range	*M*	*SD*	Range	*M*	*SD*	Range
Cluster 1 (n = 16)	48.00	9.06	40^a^–60	43.50	4.59	40^a^–56	59.69	5.94	52^b^–76	44.00	5.56	40^a^–58
Cluster 2 (n = 22)	57.82	8.48	44–71	51.91	6.69	40^a^–66	68.50	6.91	59–87	62.64	6.11	49–76
Cluster 3 (n = 21)	70.24	4.70	58–79	62.76	6.02	48–72	78.86	6.21	65–89	78.10	6.08	66–89
Cluster 4 (n = 13)	86.00	11.05	73–107	77.85	11.05	64–96	91.92	6.41	81–106	92.85	8.24	83–110

*N* = 72. *WIAT-III* Wechsler Individual Achievement Test-III, *SS* standard score.

^a^ Lowest possible standard score. ^b ^Lowest possible standard score at age 9 years.

The most striking difference between clusters was in the average level of academic achievement. Mean SSs were in the moderate disability range for three of the achievement measures and the mild disability range for one measure in Cluster 1, in the moderate disability range for one measure and the mild disability range for three measures in Cluster 2, in the mild disability range for one measure and the borderline range for three measures in Cluster 3, and in the borderline range for one measure, the low average range for one measure, and the average range for two measures in Cluster 3. As shown in Table 4, for each of the achievement measures, mean SS reliably increased between adjacent pairs of clusters, with mean standardized differences ranging from medium (five differences) to large (six differences) to very large (one difference).

**Table 4:** Pairwise group comparisons and standardized differences between adjacent clusters for each WIAT-III math and reading achievement measure.

Comparisons			Differences in SSs					Standardized differences in SSs				
A	B	Direct. Diff.	Posterior estimates		95% *CrI* for *M* Diff.		*PrD*	Posterior estimates		95% *CrI* for *M* Diff.		**Size**^**a**^
			*M* Diff.	*SD*	LL	UL		*M* Diff.	*SD*	LL	UL	
Numerical Operations SS												
C1	C2	A < B	9.84	2.82	4.33	15.39	1.00	0.66	0.19	0.28	1.02	++
C2	C3	A < B	12.41	2.64	7.21	17.60	1.00	0.83	0.18	0.48	1.17	+++
C3	C4	A < B	15.78	3.05	9.81	21.73	1.00	1.05	0.20	0.66	1.45	+++
Math Problem Solving SS												
C1	C2	A < B	8.42	2.43	3.64	13.15	1.00	0.56	0.16	0.24	0.88	++
C2	C3	A < B	10.85	2.25	6.42	15.28	1.00	0.72	0.15	0.43	1.02	++
C3	C4	A < B	15.09	2.62	9.94	20.22	1.00	1.01	0.17	0.67	1.36	+++
Basic Reading Composite												
C1	C2	A < B	8.82	2.19	4.50	13.13	1.00	0.59	0.15	0.30	0.87	++
C2	C3	A < B	10.36	2.05	6.33	14.38	1.00	0.69	0.14	0.42	0.96	++
C3	C4	A < B	13.08	2.37	8.43	17.73	1.00	0.87	0.16	0.56	1.18	+++
Reading Comprehension SS												
C1	C2	A < B	18.64	2.19	14.33	22.96	1.00	1.24	0.15	0.95	1.52	++++
C2	C3	A < B	15.47	2.03	11.48	19.47	1.00	1.03	0.14	0.76	1.29	+++
C3	C4	A < B	14.75	2.37	10.08	19.39	1.00	0.98	0.16	0.67	1.29	+++

*N* = 72 (*n*_*C1*_ = 16, *n*_*C2*_ = 22, *n*_*C3*_ = 21, *n*_*C4*_ = 13). *WIAT-III* Wechsler Individual Achievement Test-III, *SS* standard score, *Direct. Diff*. direction of difference, *M Diff.* mean difference, *PrD* probability of direction (adjusted threshold = .9875), *CrI* highest density credible interval, *Size* point estimate-based qualitative category for the size of standardized mean difference.

^a ^++ = medium, +++ = large, ++++ = very large mean difference.

### Individual-level discrepancies

#### Full-sample individual discrepancies

The patterns of discrepancy direction and statistical significance for each pair of subtests for the full sample are shown in the first section (“Overall”) of Table 5. Although Numerical Operations SS was higher than Math Problem Solving SS for most participants, this advantage was statistically significant for fewer than one-third. For comparisons between math and reading achievement, between 68 and 90% of participants earned a higher SS on the reading measure. These differences were statistically significant for more than half the participants for Basic Reading Composite and more than one-quarter for Reading Comprehension. At the same time, it is important to note that a small proportion of participants (6–10%) scored significantly higher on Numerical Operations than on one or both reading measures. This group included the three children who scored the highest on Numerical Operations (SSs from 100 to 107), all of whom had Basic Reading Composite SSs in the low average to average range (all significantly lower than Numerical Operations SSs) and Reading Comprehension SSs in the same range (two of three significantly lower than Numerical Operations SS).

**Table 5:** Relations between standard scores on mathematics and reading measures for individual participants: patterns of nominal and significant differences.

Measures		Observed (nominal) discrepancy							Significant discrepancy (*p* < 0.05)			
A	B	A > B			A = B	A < B			CV	A > B	NS	A < B
		%	*M* Diff.	*SD*	%	%	*M* Diff.	*SD*		%	%	%
Overall (*N* = 72)												
NOP	MPS	72.3	10.44	7.14	8.3	19.4	5.57	4.23	11	25.0	73.6	1.4
	BRC	16.7	7.58	5.95	1.4	81.9	13.07	7.34	9	4.2	43.1	52.7
	RC	25.0	10.28	6.46	6.9	68.1	9.82	6.87	12	9.7	66.7	23.6
MPS	BRC	5.6	4.00	2.16	4.2	90.2	17.86	8.26	9	0.0	19.4	80.6
	RC	12.5	6.22	4.71	8.3	79.2	14.33	7.71	12	1.4	59.7	38.9
*Clusters*												
Cluster 1 (*n* = 16)												
NOP	MPS	43.8	13.43	8.04	25.0	31.2	4.40	2.61	11	18.8	81.2	0.0
	BRC	6.2	2.00	0.00	6.2	87.6	13.50	6.44	9	0.0	31.2	68.8
	RC	43.8	12.43	8.62	18.8	37.4	3.83	3.37	12	18.8	81.2	0.0
MPS	BRC	0.0	0.00	0.00	0.0	100.0	16.19	7.46	9	0.0	12.5	87.5
	RC	37.5	5.33	5.24	25.0	37.5	6.67	3.39	12	6.2	93.8	0.0
Cluster 2 (*n* = 22)												
NOP	MPS	72.7	10.31	6.69	4.6	22.7	7.00	5.96	11	31.8	63.6	4.6
	BRC	18.2	6.75	3.86	0.0	81.8	14.56	9.00	9	0.0	50.0	50.0
	RC	31.8	8.86	3.29	4.6	63.6	12.00	6.91	12	9.1	63.6	27.3
MPS	BRC	4.5	4.00	0.00	4.5	91.0	18.45	9.91	9	0.0	22.7	77.3
	RC	9.1	10.00	1.41	4.5	86.4	13.47	7.53	12	0.0	68.2	31.8
Cluster 3 (*n* = 21)												
NOP	MPS	85.7	8.94	6.29	4.8	9.5	2.00	1.41	11	23.8	76.2	0.0
	BRC	14.3	3.00	2.65	0.0	85.7	10.56	6.37	9	0.0	57.1	42.9
	RC	9.5	3.00	1.41	0.0	90.5	9.00	6.90	12	0.0	66.7	33.3
MPS	BRC	4.8	1.00	0.00	0.0	95.2	16.95	7.81	9	0.0	14.3	85.7
	RC	0.0	0.00	0.00	0.0	100.0	15.33	6.54	12	0.0	38.1	61.9
Cluster 4 (*n* = 13)												
NOP	MPS	84.6	11.18	8.72	0.0	15.4	8.50	0.71	11	23.1	76.9	0.0
	BRC	30.8	13.25	5.91	0.0	69.2	14.44	6.60	9	23.1	23.1	53.8
	RC	15.4	15.00	0.00	7.7	76.9	11.90	6.61	12	15.4	53.8	30.8
MPS	BRC	15.4	5.50	0.71	15.4	69.2	21.56	7.38	9	0.0	30.8	69.2
	RC	7.7	4.00	0.00	7.7	84.6	18.09	9.22	12	0.0	38.5	61.5

*M Diff*. mean difference, *CV* critical value for a statistically significant discrepancy (at the *p* < 0.05-level) for an individual child between SSs for the specified pair of measures at age 9 years as reported in the Wechsler Individual Achievement Test-III technical manual, *%* percentage of participants who evidenced a specific discrepancy direction, *NS* nonsignificant discrepancy.

*NOP* Weschler Individual Achievement Test-III (WIAT-III) Numerical Operations standard score (SS), *MPS* WIAT-III Math Problem Solving SS, *BRC* WIAT-III Basic Reading Composite, *RC* WIAT-III Reading Comprehension SS.

#### Cluster-level individual discrepancies

In the second part of Table 5 (“Clusters”), the patterns of discrepancy direction and statistical significance for each pair of subtests are shown separately for the individuals in each of the four clusters. Most of the patterns of individual discrepancies were similar across clusters. For example, between 19 and 32% of the participants in each cluster scored significantly higher on Numerical Operations than on Math Problem Solving; for all but one of the remaining participants, SSs on the two math measures did not differ significantly although most of the nominal differences favored Numerical Operations. Across clusters, Basic Reading Composite SSs were significantly higher than Numerical Operations SS for 43–69% of participants and significantly higher than Math Problem Solving SS for 69–88% of participants. The proportion of participants who earned significantly higher Reading Comprehension SSs than Numerical Operations SS was considerably lower and did not include any participants in cluster 1. The most discrepant results between clusters were for the relations between Math Problem Solving SS and Reading Comprehension SS: for this comparison, 62% of the participants in each of Clusters 3 and 4 scored significantly higher on Reading Comprehension, with a considerably lower proportion (32%) for Cluster 2 and 0% for Cluster 1.

Very few children scored significantly higher on the math measures than on the reading measures. All three who scored significantly higher on Numerical Operations than Basic Reading Composite were in Cluster 4; the seven who scored significantly higher on Numerical Operations than Reading Comprehension were in Clusters 1, 2, and 4. For Math Problem Solving, no participant scored significantly higher than on Basic Reading Composite and only one child (Cluster 1) scored significantly higher than on Reading Comprehension.

### Multiple regression analyses

Bayesian bivariate correlations among the variables used in the regression analyses are reported in Table 6. All correlations had decisive evidence in their support (*BF*_*10*_ > 10^2^).

**Table 6:** Bayesian Pearson correlations among variables used in the regression analyses.

Variable	2	3	4	5	6
1. Verbal SS	0.55	0.47	0.70	0.54	0.67
2. Nonverbal Reasoning SS		0.59	0.63	0.60	0.68
3. Spatial SS			0.56	0.56	0.57
4. Working Memory SS				0.74	0.75
5. Numerical Operations SS					0.78
6. Math Problem Solving SS					

*N* = 72. *SS* standard score. Bayes factors for the coefficients ranged from 10^3^ to 10^14^, indicating decisive evidence for the existence of a linear association between every pair of variables included in the table.

To investigate the cognitive predictors of mathematical performance in computation and problem solving, two Bayesian multiple linear regressions were fitted, with WIAT-III Numerical Operations and Math Problem Solving SSs as the dependent variables and DAS-II Verbal, Nonverbal Reasoning, Spatial, and Working Memory SSs as the independent variables. Model coefficients, support intervals, and *BF*s are presented in Table 7.

**Table 7:** Bayesian multiple linear regression predicting WIAT-III Numerical Operations and Math Problem Solving standard scores.

Model	Posterior estimates		BF = 3.16 *SI *for *M*		Bayes factor (*BF*_*10*_)	
	*M*	*SD*	LL	UL	Estimate	Strength^a^
Numerical Operations SS						
(Constant)	64.33	1.15	62.62	66.15	—	—
Verbal SS	0.00	0.11	–0.09	0.08	9.50 × 10^–1^	–
Nonverbal Reasoning SS	0.19	0.13	0.11	0.33	1.37 × 10	++
Spatial SS	0.18	0.11	0.08	0.30	1.79 × 10	++
Working Memory SS	0.49	0.11	0.35	0.66	7.59 × 10^4^	+++
*R*^*2*^	0.60	0.10	—	—	—	—
Math Problem Solving SS						
(Constant)	57.88	0.90	56.42	59.43	—	—
Verbal SS	0.19	0.09	0.09	0.32	5.62 × 10	++
Nonverbal Reasoning SS	0.31	0.11	0.19	0.47	4.32 × 10^2^	+++
Spatial SS	0.09	0.09	–0.02	0.21	5.87	+
Working Memory SS	0.30	0.09	0.18	0.43	1.99 × 10^3^	+++
*R*^*2*^	0.71	0.09	—	—	—	—

*N* = 72. *WIAT-III* Wechsler Individual Achievement Test-III, *SI* support interval, *BF* Bayes factor, *SS* standard score.

^a ^− = negligible evidence for the null hypothesis, + = substantial, ++ = strong, +++ = decisive evidence for the experimental hypothesis.

#### Numerical Operations

The point estimate from the posterior distribution of *R*^*2*^*s* indicated that the Numerical Operations model explained 60% of the observed variance in SSs on this measure. The nominally higher *R*^*2*^ estimate compared to the value reported for typically developing individuals by Hale et al.^19^ is consistent with the higher bivariate correlations for the present participants. Null-interval *BF*s indicated strong (Nonverbal Reasoning, Spatial SS) to decisive (Working Memory SS) evidence in favor of unique contributions to the variance in Numerical Operations SS and negligible evidence in favor of the null hypothesis regarding the contribution of Verbal SS. Using the posterior point-estimates for this model, a 1-point increase in Working Memory SS results in a 0.49-point increase; a 1-point increment in Nonverbal Reasoning SS results in a 0.19-point increase; and a 1-point increase in Spatial SS results in a 0.18-point increase in Numerical Operations SS with the remaining predictors held constant.

#### Math Problem Solving

The point-estimate from the posterior distribution of *R*^*2*^*s* indicates that this model explains 71% of the variance in Math Problem Solving SS. As in the Numerical Operations model and consistent with the bivariate correlations among variables included in the model, the *R*^*2*^ was nominally higher than the estimate reported by Hale et al.^19^ for typically developing children. Evidence supporting a unique contribution to the variance in Math Problem Solving SS was decisive for Nonverbal Reasoning SS and Working Memory SS, strong for Verbal SS, and substantial for Spatial SS. Based on the posterior point estimates, after controlling for the effect of the remaining predictors, a 1-point increase in Nonverbal Reasoning SS results in a 0.31-point increase; a 1-point increase in Working Memory SS results in a 0.30-point increase; a 1-point increase in Verbal SS results in a 0.19-point increase; and a 1-point increase in Spatial SS results in a 0.09-point increase in Math Problem Solving SS.

## Discussion

In this study, we analyzed the math achievement of 9-year-olds with WS, including average levels, full range, comparison to reading achievement, and cognitive predictors. We found wide variability in math achievement among children with WS, with average levels lower than those for reading achievement. The cognitive predictors of math achievement were consistent with those for typically developing individuals. We discuss these results below.

### Math achievement among individuals with Williams syndrome

For both computation and word problem solving, the average levels of functioning were in the mild disability range. Most individuals scored in the borderline to moderate disability range, but the full range extended from a lower extreme of severe disability to an upper extreme of average ability for the general population. Although the average Numerical Operations and Math Problem Solving SSs were consistent with scaled scores reported in previous studies using the Arithmetic subtest from the WISC or WAIS^32,33^, the variability in scores in our sample was considerably higher and comparable to that for the general population. The median math AEs for the present sample also were consistent with the AEs reported in prior studies^35,38^. Similarly, the highest AEs reported for the present sample were consistent with the highest AEs reported in previous studies. However, these apparent consistencies are misleading because the participants in our study were considerably younger than those in the previous studies, both of which included adults in addition to school-age children. In contrast to prior studies, for which the AEs were much younger than expected for the participants’ chronological ages, for many of the participants in the current study, the obtained AEs corresponded to average performance for their chronological age (as indicated by SSs in the low average to average range).

We expected reading achievement to be stronger than math achievement based on findings from an independent sample of participants in our lab using the WIAT-II (see Table S1). As hypothesized, at the group level, there was decisive evidence that math achievement was lower than reading achievement. These group-level differences were further reflected in statistically significant differences at the individual level, especially for comparisons involving word reading, with 81% of the participants showing a statistically significant discrepancy favoring Basic Reading Composite in comparison to Math Problem Solving and 57% in comparison to Numerical Operations. Significant differences at the individual level were less common for comparisons involving Reading Comprehension. In addition, as hypothesized, there was decisive evidence that math computation performance was stronger than math problem solving performance.

Findings from the cluster analysis indicated that the primary difference between adjacent clusters was in overall level of academic achievement, with average performance ranging from predominantly in the moderate disability range for Cluster 1 to predominantly in the low average to average range for Cluster 4. A similar pattern of discrepancies between Numerical Operations SS and Math Problem Solving SS was shown by all four clusters, and patterns of discrepancies between performance on the math subtests and the reading subtests were largely consistent across clusters.

### Concurrent cognitive predictors of math achievement standard scores in individuals with Williams syndrome

Based on findings from several meta-analyses^10,14,15^ and Detterman and Daniel's^11^ study, we hypothesized that both math computation and math problem solving would be correlated with each of the cognitive predictors (verbal, nonverbal reasoning, spatial, and working memory ability) examined in the current study. In line with this hypothesis, there was decisive evidence favoring the existence of a positive correlation between both measures of math achievement and each of the cognitive ability variables. These correlations with math achievement were similar in magnitude to the few correlations previously reported for individuals with WS for verbal ability^38^ and nonverbal reasoning ability^35,38^ and somewhat stronger than the correlation previously reported for spatial ability^35^. The correlation coefficients in the present study were higher than the meta-analytic correlations reported by Peng et al.^10,14,15^, corroborating Detterman and Daniel's^11^ findings of stronger correlations between cognitive and achievement measures among individuals with IQs in the borderline or ID range.

As indicated previously, we used the proportion of variance explained by DAS-II predictors reported by Hale et al.^19^ as the minima for modeling the proportion of variance explained by our models. We based this decision on Detterman and Daniel's^11^ findings reporting stronger correlations among cognitive abilities in the lower IQ group compared to the average IQ group for both the WISC and the WAIS. In addition, we relied on Villeneuve et al.'s^16^ bifactor model proportion of variance estimates, which indicated stronger multiple correlations for the math problem solving model compared to the math computation model. In line with our initial hypothesis, we found that the linear combination of concurrent cognitive predictors of math achievement in this study explained a large proportion of the variance observed in WIAT-III Numerical Operations SS (60%) and Math Problem Solving SS (71%). Both values were higher than the point estimates reported by Hale et al.^19^ for the DAS-II–WIAT-II linking sample (ages 6–17 years) and Villeneuve et al.^16^ for children in Grades 3–4.

In the model predicting WIAT-III Numerical Operations SS, we found decisive evidence favoring an effect of DAS-II Working Memory SS and strong evidence for effects of Spatial and Nonverbal Reasoning SSs. In contrast, the unique contribution of DAS-II Verbal SS was negligible. These findings contrast with Hale et al.’s^19^ finding that Verbal SS accounted for a significant proportion of unique variance in WIAT-II Numerical Operations SS but are consistent with Villeneuve et al.’s^16^ bifactor model showing that verbal reasoning did not have a statistically significant direct contribution to math computation.

In the model predicting WIAT-III Math Problem Solving SS, there was decisive evidence for an effect of DAS-II Working Memory and Nonverbal Reasoning SSs. Evidence supporting an effect of Verbal SS was strong, while evidence for an effect of Spatial SS was substantial. The difference in contributions of Nonverbal Reasoning SS and Spatial SS is consistent with Peng et al.’s^14^ finding that nonverbal reasoning was more strongly related than visuospatial reasoning to overall math achievement. Once more, these findings corroborate the results reported by Villeneuve et al.^16^ for the bifactor model. Although we found relatively larger unique contributions of Nonverbal Reasoning and Working Memory SSs and a relatively smaller contribution of Spatial SS than reported by Hale et al.^19^, our findings are consistent with theirs in that both studies found evidence that Verbal, Nonverbal Reasoning, Spatial, and Working Memory SSs each contributed unique variance to math problem solving ability.

### Implications

In the present study, the strongest cognitive predictor of math achievement for children with WS was working memory ability, which typically was in the mild intellectual disability range. In this context, working memory training might seem to be a logical approach to improving math achievement. However, the findings of meta-analyses^51,52^ of more than two decades of research examining working memory training as an intervention to increase academic achievement for individuals with cognitive or learning difficulties make it clear that working memory training is not the solution; effect sizes for transfer to academic achievement are very small or null. Thus, a different approach is needed. Hattie^53^ as well as Hattie et al.^54^, based on a synthesis of a large number of meta-analyses, have identified a number of effective strategies for increasing the math achievement of individuals in the general population or individuals with learning or cognitive disabilities. Among these strategies, approaches that reduce cognitive load by limiting working memory demands (especially approaches that also support nonverbal reasoning, another strong predictor of math achievement for children with WS) are most likely to be effective. Direct instruction providing detailed, step-by-step demonstrations of problem solving and timely corrective feedback is crucial. A set of worked examples available to the child at their desk showing all the steps needed to arrive at the correct answer for each of the types of problems that are being focused on in the classroom would provide further support, allowing the child to focus on the concepts being taught. Manipulatives and number lines are likely to facilitate initial understanding, identification of patterns, and generalization of mathematical concepts. The other important predictor for math problem solving for children with WS was verbal ability. In addition to speech-language therapy focused on increasing comprehension of complex grammatical structures (necessary to understand classroom discourse and the wording of math story problems) and classroom instruction focused on expanding general vocabulary, building math vocabulary—especially quantitative and spatial relational terms (an area of specific weakness for children with WS^27^) is crucial both to allow the child to benefit from direct instruction and the use of manipulatives and to help the child understand the math problem to be solved and then identify which of the worked examples is the appropriate model for that problem.

### Limitations and future directions

In interpreting our results, one must consider several limitations. Despite our best efforts, although our sample included participants from all US census regions and two Canadian provinces, the sample was composed predominantly of White non-Hispanic individuals. In addition, the current study is cross-sectional and included only 9-year-olds. Both additional cross-sectional studies including children in other age ranges and longitudinal studies would be helpful in elucidating variation and growth in math achievement in individuals with WS.

## Conclusions

The average math achievement of 9-year-olds with WS in this study was in the mild disability range, with the full range of achievement including extremes of severe disability to average ability for the general population, a considerably wider range than suggested by previous research. At the same time, the cognitive predictors of math achievement—working memory, nonverbal reasoning, verbal reasoning, and spatial reasoning—were similar to those for typically developing individuals. Given these similarities, effective teaching strategies identified in meta-analyses such as direct instruction, provision of worked examples as models, use of manipulatives, and relational vocabulary instruction^53,54^, have the potential to meaningfully increase the math achievement of individuals with WS.

### Supplementary Information

Supplementary Information.

## Data Availability

The data that support the findings of this study are available from the corresponding author upon reasonable request.
