# Supplementary material for: Concurrent predictors of mathematics achievement for 9-year-old children with Williams syndrome
Source: Sci Rep. 2024 Jan 30;14:2454. doi: 10.1038/s41598-024-52639-7 (PMC10827727; doi:10.1038/s41598-024-52639-7)
Supplement: Supplementary file 1 — Supplementary Information. [file 41598_2024_52639_MOESM1_ESM.pdf]

Supplementary Materials for:

Concurrent Predictors of Mathematics Achievement for 9-year-old Children with Williams Syndrome

Vitor N. Guimaraes and Carolyn B. Mervis

Appendix A: WIAT-II Mathematics and Reading Achievement Performance

For the second research question, our hypotheses were based in part on previously unpublished data from our laboratory addressing performance of an independent sample of children with Williams syndrome (WS) on the Wechsler Individual Achievement Test–second edition (WIAT-II) [1]. Below, we describe the participants and the WIAT-II subtests administered and then present descriptive statistics for performance on these subtests.

Participants and data collection. The WIAT-II sample included 46 children (19 boys, 27 girls) with genetically-confirmed classic length WS microdeletions aged 9.09 – 14.70 years (*M* = 11.60 years, *SD* = 1.88). All participants were native speakers of English. They resided in 20 U.S. states representing all census regions (36.9% Midwest, 28.3% South, 19.6% Northeast, 15.2% West). The racial/ethnic background of the participants was 89.1% White non-Hispanic, 8.7% White Hispanic, and 2.2% Black/African American non-Hispanic.

Participants were administered the mathematics and reading subtests from the WIAT-II [1] as part of a broader assessment by trained examiners. Data collection started in February 2007 and ended in August 2009.

Measures. Of relevance for the current study were the WIAT-II Numerical Operations, Math Reasoning, Word Reading, Pseudoword Decoding, and Reading Comprehension subtests. All measures used yielded standard scores (SSs) with a general population mean of 100 (*SD* = 15). The average of the manual-reported split-half internal consistency estimates for each of the intervals from ages 9 – 14 years

[1] was .92 for Numerical Operations, .94 for Math Reasoning, .97 for Word Reading, .97 for Pseudoword Decoding, and .96 for Reading Comprehension.

The WIAT-II Math Reasoning, Numerical Operations, and Pseudoword Decoding subtests were updated for the WIAT-III (the version of the WIAT used in the study reported in the main text) but cover the same content. (The WIAT-II Math Reasoning subtest was renamed Math Problem Solving for the WIAT-III.) However, although the names of the Word Reading and Reading Comprehension subtests remained the same on the WIAT-II and the WIAT-III, the content changed considerably. The WIAT-II Word Reading subtest included a large number of items testing letter-name knowledge and phonological awareness in addition to single words that the child was asked to read aloud. In contrast, the WIAT-III Word Reading subtest included only single words to be read aloud. (The WIAT-III included a new subtest – Early Reading – that addressed letter-name knowledge and phonological awareness, along with a few items measuring sight-word knowledge.) The WIAT-II Reading Comprehension subtest included items for which the child was asked to select the picture that matched a written word, items for which the child read a sentence and then answered a question about it, and items for which the child read a passage and then answered questions about it. In contrast, the WIAT-III Reading Comprehension subtest was composed entirely of passages that the child read and then answered questions about.

Results. Descriptive statistics for SSs on the five WIAT-II subtests are shown in Table S1. There was a wide range of performance on each subtest, with median SS in the mild disability range for the math subtests and in the borderline range for the reading subtests. For all five subtests, the full range of SSs was from moderate/severe disability to average ability for the general population.

As indicated in the table, average SSs for Numerical Operations and Math Reasoning were nominally lower than average SSs on the reading subtests. No inferential statistical comparisons were made because the type of content included in two of the reading subtests changed substantially between the WIAT-II and WIAT-III so the WIAT-II findings would not be useful in making specific

predictions regarding performance on the WIAT-III math subtests relative to the reading subtests, beyond providing further evidence for the finding reported in the Introduction to the main text that math performance did not exceed reading performance. Moreover, average Math Reasoning SS was slightly lower compared to average Numerical Operations SS. This finding provided the basis for our hypothesis that, in the main study, the average SS for Numerical Operations would be higher than the average SS for Math Problem Solving.

Table S1

Descriptive Statistics for WIAT-II Mathematics and Reading Measures

Measures	Mean	Median	*SD*	IQR	Range
Mathematics					
Numerical Operations SS	60.89	58.00	15.49	50.00–67.25	40[c]–102
Math Reasoning[a] SS	57.00	56.00	15.16	42.00–65.08	40[c]–94
Reading					
Word Reading SS	73.04	76.50	20.81	52.92–88.08	40[c]–108
Pseudoword Decoding SS	79.17	77.50	15.33	69.00–90.08	40[c]–110
Mean Word – Pseudoword[b] SS	75.87	77.00	17.68	61.92–89.08	40[c]–106
Reading Comprehension SS	65.50	68.00	19.34	45.75–82.17	40[c]–102

Note. *N* = 46. WIAT-II = Wechsler Individual Achievement Test-II, SS = standard score.
[a] Equivalent to WIAT-III Math Problem Solving. [b] Mean of Word Reading and Pseudoword Decoding SSs used as a measure similar to WIAT-III Basic Reading Composite. [c] Lowest possible standard score.

Appendix B: Sampled Prior Distributions Used in the Inferential Analyses

Descriptive statistics for the sampled prior distributions used in all analyses are presented in Table S2 below. The table is divided into four panels corresponding to the main families of analyses conducted: robust estimation, multivariate linear model post-hoc comparisons (i.e., comparisons between adjacent clusters), bivariate correlations, and multiple linear regressions.

In the robust estimation family, comparisons between the Wechsler Individual Achievement Test–third edition (WIAT-III) [2] math and reading achievement measures were conducted. The parameters of the distributions of differences for the WIAT-III math and reading achievement comparisons were based on information provided in Table S1 above, as described in the Data Analysis section of the manuscript. For the DAS-II comparisons, the distributions of differences were based on results reported by Mervis and John [3] for an independent sample of individuals. The cluster analysis was a study-specific approach to investigating the possibility of discrepant patterns of achievement within the sample of the present study. For this reason, no sufficiently informative priors could be derived from the literature. Instead, we opted for flat priors having their lower and upper limits set at the minima and maxima of differences between clusters as per observed data.

For the correlation analyses, no specific prior beliefs were expressed, and weakly-informative beta priors were set. For the regression analyses, priors were set on the amount of explained variance by the linear combination of predictors. Two sources of information were used to derive prior distributions: Villeneuve and colleagues [4] and, more importantly, Hale and colleagues [5].

Table S2

Descriptive Statistics for the Prior Distributions Used in All Inferential Analyses

Analyses/Parameters	*M*	*SD*	*Mdn*	Interquartile Range		95% HDI		Skewness	Kurtosis
				25th	75th	LL	UL		
Robust Estimation									
WIAT-III									
NOP x MPS SS									
(Constant)	63.86	13.59	62.67	53.58	72.70	40.00	87.97	0.54	0.10
Difference	–3.87	11.82	–3.87	–11.63	3.92	–27.08	19.62	–0.01	0.20
Error	16.52	20.03	11.43	5.28	21.42	0.00	47.50	9.59	318.20
NOP x BRC SS									
(Constant)	63.84	13.53	62.65	53.63	72.60	40.01	87.94	0.54	0.11
Difference	12.16	14.55	12.19	2.60	21.78	–16.42	40.72	0.00	0.22
Error	16.46	19.50	11.55	5.29	21.14	0.00	47.54	7.25	171.70
NOP x RC SS									
(Constant)	63.87	13.54	62.76	53.63	72.68	40.01	87.75	0.53	0.09
Difference	7.25	16.24	7.22	–3.53	18.03	–25.01	39.06	0.01	0.21
Error	16.67	19.90	11.59	5.33	21.41	0.00	48.05	6.89	123.30
MPS x BRC SS									
(Constant)	61.07	12.68	59.74	51.36	69.12	40.00	84.02	0.63	0.25
Difference	16.80	14.21	16.78	7.39	26.25	–10.93	44.90	–0.01	0.19
Error	16.50	19.44	11.42	5.18	21.43	0.00	47.81	6.35	118.50
MPS x RC SS									
(Constant)	60.90	12.67	59.51	51.16	68.98	40.00	83.91	0.64	0.24
Difference	11.24	14.21	11.30	1.84	20.64	–17.08	39.01	–0.02	0.24
Error	16.53	19.76	11.41	5.24	21.29	0.00	47.54	7.36	171.60
DAS-II									
Verbal x Spatial SS									
(Constant)	74.42	16.47	74.22	63.32	85.29	41.45	106.30	0.13	0.01
Difference	–19.27	15.04	–19.23	–29.16	–9.40	–49.07	10.47	–0.01	0.25
Error	16.48	19.80	11.49	5.27	21.35	0.00	47.49	10.46	396.50

Table S2 (continued)

Descriptive Statistics for the Prior Distributions Used in all Inferential Analyses

Analyses/Parameters	*M*	*SD*	*Mdn*	Interquartile Range		95% HDI		Skewness	Kurtosis
				25th	75th	LL	UL		
NV Reasoning x Spatial SS									
(Constant)	79.03	15.79	79.02	68.55	89.54	47.81	110.10	0.05	0.07
Difference	–24.08	14.06	–24.09	–33.39	–14.78	–51.48	3.71	0.01	0.21
Error	16.60	19.26	11.58	5.28	21.50	0.00	47.55	5.25	63.33
Cluster Comparisons									
Numerical Operations SS									
(Constant)	63.85	13.55	62.68	53.54	72.66	40.00	87.92	0.53	0.06
Difference	–0.03	38.69	0.09	–33.55	33.63	–66.36	60.78	0.00	–1.20
Error	16.74	21.53	11.55	5.27	21.42	0.00	48.17	17.19	1175.00
Math Problem Solving SS									
(Constant)	60.97	12.72	59.56	51.13	69.02	40.01	84.05	0.64	0.25
Difference	–0.07	32.30	–0.12	–28.01	27.86	–51.79	54.48	0.00	–1.20
Error	16.68	20.05	11.55	5.29	21.45	0.00	48.55	9.15	333.80
Basic Reading Composite									
(Constant)	79.17	15.44	77.86	67.42	89.21	52.00	106.60	0.52	0.04
Difference	–0.01	31.20	–0.02	–27.10	27.01	–53.03	49.47	0.00	–1.20
Error	16.61	19.99	11.49	5.24	21.43	0.00	47.93	7.66	181.80
Reading Comprehension SS									
(Constant)	69.36	16.87	67.86	56.52	80.27	40.00	99.42	0.54	0.05
Difference	–0.18	40.44	–0.14	–35.20	34.73	–67.33	65.67	0.01	–1.20
Error	16.55	20.29	11.41	5.29	21.22	0.00	47.52	8.10	189.80
Residual Correlation	0.00	0.45	0.00	–0.35	0.35	–0.81	0.81	0.00	–0.86
Bivariate Correlations									
Correlation Coefficient	0.50	0.19	0.50	0.36	0.64	0.15	0.86	–0.01	–0.67

Table S2 (continued)

Descriptive Statistics for the Prior Distributions Used in Inferential all Analyses

Analyses/Parameters	*M*	*SD*	*Mdn*	Interquartile Range		95% HDI		Skewness	Kurtosis
				25th	75th	LL	UL		
Regressions									
Numerical Operations SS									
(Constant)	63.96	13.59	62.80	53.65	72.81	40.00	88.06	0.53	0.08
R^2	0.66	0.10	0.66	0.59	0.73	0.47	0.85	–0.29	–0.11
Error	16.52	18.99	11.48	5.27	21.34	0.00	48.06	5.53	83.87
Math Problem Solving SS									
(Constant)	61.09	12.76	59.75	51.29	69.19	40.00	84.12	0.63	0.25
R^2	0.77	0.09	0.78	0.72	0.84	0.60	0.93	–0.53	0.13
Error	16.60	19.97	11.54	5.24	21.46	0.00	47.65	8.39	250.10

Note. N (random samples) = 40,000. HDI = highest density interval, SS = standard score, NOP = Wechsler Individual Achievement Test-III (WIAT-III) Numerical Operations, MPS = WIAT-III Math Problem Solving, BRC = WIAT-III Basic Reading Composite, RC = WIAT-III Reading Comprehension, NV Reasoning = DAS-II Nonverbal Reasoning.

Appendix C: Nonparametric Descriptive Statistics for Wechsler Individual Achievement Test—third edition (WIAT-III) Math Achievement Age Equivalents and Standard Scores

Table S3

Nonparametric Descriptive Statistics for Age Equivalents and Standard Scores for WIAT-III Mathematical Skill Measures

Measure	Median	IQR	Range
Age Equivalent (Years)			
Numerical Operations	6.34	5.34–7.00	<5.00[a]–9.67
Math Problem Solving	6.00	5.34–6.67	<4.00[a]–8.67
Standard Score			
Numerical Operations	67.00	52.00–73.00	40[b]–107
Math Problem Solving	58.00	48.00–66.00	40[b]–96

Note. *N* = 72. IQR = interquartile range.

[a] Lowest possible age equivalent for the subtest, [b] Lowest possible standard score.

References

1. Wechsler, D. *Wechsler Individual Achievement Test–second edition (WIAT-II), update 2005: Manual*. (Psychological Corporation, 2005).
2. Wechsler, D. *Wechsler Individual Achievement Test–third edition (WIAT-III): Manual*. (Pearson, 2009).
3. Mervis, C. B. & John, A. E. Cognitive and behavioral characteristics of children with Williams syndrome: Implications for intervention approaches. *Am. J. Med. Genet. C* **154C**, 229–248 (2010).
4. Villeneuve, E. F., Hajovsky, D. B., Mason, B. A. & Lewno, B. M. Cognitive ability and math computation developmental relations with math problem solving: An integrated, multigroup approach. *School Psychol.* **34**, 96–108 (2019).
5. Hale, J. B. *et al.* Differential ability scales–second edition (neuro)psychological predictors of math performance for typical children and children with math disabilities. *Psychol. Schools* **45**, 838–858 (2008).
